# *Ptychoptera deleta* Novák, 1877 from the Early Miocene of the Czech Republic: redescription of the first fossil attributed to Ptychopteridae (Diptera)

**DOI:** 10.3897/zookeys.130.1401

**Published:** 2011-09-24

**Authors:** Wieslaw Krzemiński, Jakub Prokop

**Affiliations:** 1 Institute of Biology, Pedagogical University of Cracow, ul. Podbrzezie 3, 31-054 Kraków, Poland; 2 Charles University in Prague, Faculty of Science, Department of Zoology, Viničná 7, CZ-128 44, Praha 2, Czech Republic

**Keywords:** Diptera, Ptychopteridae, Ptychoptera, revision, Tertiary, Neogene, Miocene, Ottnangian/Karpatian, Cypris Formation, Cheb Basin, Czech Republic

## Abstract

The first fossil that was described in Ptychopteridae, *Ptychoptera deleta*Novák, 1877 from the classical Early Miocene locality Mokřina (Krottensee) in western Bohemia is re-examined. The re-description of the holotype including a new line drawing and remarks summarizing the scarce fossil record of this group is provided.

## Introduction

The family Ptychopteridae is a very small group with about 70 Recent species belonging to two subfamilies (Ptychopterinae and Bittacomorphinae), mostly distributed in the Holarctic, Ethiopian and Oriental Regions. In the Neotropics one species is currently known (Hancock et al. 2006). Ptychopteridae are not present in the Australian and Antarctic Regions. The oldest true representative, *Ptychoptera mesozoica* Kalugina, 1989 was described from the Lower Cretaceous (Neocomian) of Baissa in Buryatia (Siberia, Russia). *Ptychoptera deleta* Novák, 1877, known from the Early Miocene of Mokřina (Krottensee) in western Bohemia (Czech Republic), was the first described fossil representative of the family. Handlirsch (1909) supposed that the species did not belong to the genus *Ptychoptera* Meigen, 1803 and created a new genus *Ptychopterula* mainly on the basis of a considerably narrow wing base as present in *Etoptychoptera* Handlirsch, 1909 and a free Sc. However, he emphasized also the common characters present in *Ptychopterula* and the Recent genera *Ptychoptera* and *Bittacomorpha* Westwood, 1835 connection of R and Rs, the position of crossvein r-m, etc. Alexander (1927) also supposed that the species described by Novák did not belong to *Ptychoptera*. Peus (1958), in his monograph on the Ptychopteridae, referred to this species as *Liriope* (“*Ptychopterula*”) *deleta* (Novák, 1877). These opinions were based on mistakenly Novak’s drawing only, without revision of the holotype (see Fig. 2B). This paper presents a re-description of the holotype including a new line-drawing and photograph. Five other compressed fossil ptychopterid species were described till now: *Ptychoptera miocenica* (Cockerell, 1910) from the Oligocene of Florissant (Colorado, USA) originally placed in genus *Bittacomorpha*; *Brodilka mitchelli*Lukashevich, Coram & Jarzembowski, 2001 and *Zhiganka woolgari*Lukashevich, Coram & Jarzembowski, 2001 both from the Lower Cretaceous of Purbeck and Wealden groups (UK); *Zhiganka comitans* Lukashevich, 1995 from the Lower Cretaceous of Yakutia (Russia), and *Probittacomorpha christenseni* Freiwald & Willmann, 1992 from the lowermost Eocene of Mo-clay (Denmark) (Freiwald and Willmann 1992; Evenhuis 1994; Lukashevich 1995; Ansorge and Schröder 1999; Lukashevich et al. 2001). Two genera, *Zhiganka*and*Probittacomorpha*areattributed to the subfamily Bittacomorphinae and others to Ptychopterinae. A single species, *Ptychoptera eocenica* Podenas, 2007 was described from an Eocene Baltic amber inclusion (Podenas 2007).

Lukashevich (2008) proposed a new system of Ptychopteroidea and synonymized the family Eoptychopteridae known from the Upper Triassic to Lower Cretaceous with Ptychopteridae. Eoptychopteridae is a considerably variable group of flies with common occurrence throughout the Jurassic up to the Lower Cretaceous (Kalugina 1989; Krzemiński 1992; Lukashevich 1993; Lukashevich et al. 1998; Ren and Krzemiński 2002; Krzemiński and Krzemińska 2003).

**Figure 1.**
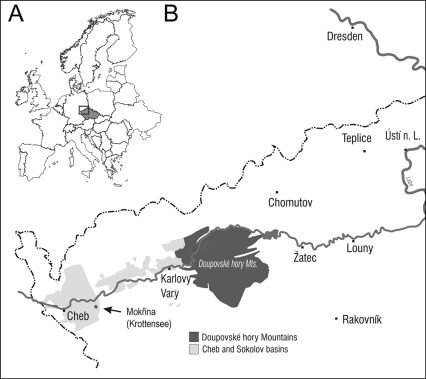
** A**Geographical position of northwestern Bohemia within Europe **B** detailed map of the Cheb and Sokolov basins with indication of position of Mokřina (Krottensee) locality.

Novák (1877) described a fossil entomofauna from Mokřina (Krottensee) located in Cheb county of western Bohemia (Czech Republic) (Fig. 1). This classical Early Miocene locality in Cheb Basin yielded greenish marls of lake sedimentation belonging to the Cypris Formation (Rojík 2004). Fossiliferous layers previously called “Cypris shales” are well known for abundant occurrence of ostracods e.g. *Cypris angusta* (Reuss, 1852) when Cheb and Sokolov basins were interconnected (Obrhelová and Obrhel 1987). Mokřina (Krottensee) locality belongs to biostratigraphic zone MN4 dated by mammals from nearby locality Dolnice (Fejfar 1974), and to ichthyozone IV defined by Obrhelová and Obrhel (1983). Fossil record from Mokřina is also well known by plants and bird remains (Bůžek et al. 1996; Mlíkovský 1996). Novák’s collection from Mokřina housed in National Museum in Prague contains 73 insect specimens classified in 10 families of five insect orders (Prokop et al. 2003).

## Material and methods

The holotype specimen was observed under stereomicroscope Leica MZFLIII & MZ16 in dry state. The line drawing of the venational pattern was drawn directly using a stereomicroscope and finally readjusted to the photograph scale using image-editing software (Adobe Photoshop CS). Photography was made simultaneously using a digital camera Canon PowerShot S80 attached to the stereomicroscope. The type material referred to as ‘NM’ is housed at the National Museum in Prague, Czech Republic.

## Systematic paleontology

### Family Ptychopteridae Osten-Sacken, 1879

#### Genus Ptychoptera

Meigen, 1803

##### Type species.

*Tipula contaminata* Linnaeus, 1758

#### Ptychoptera deleta

Novák, 1877

http://species-id.net/wiki/Ptychoptera_deleta

Figs 2A–C

Ptychoptera deleta Novák, 1877: p. 88, Pl. II, Fig.1.
Ptychopterula deleta (Novák, 1877): Handlirsch (1909): p. 264, 269.
Liriope (“*Ptychopterula*”) *deleta* (Novák, 1878): Peus (1958):p. 12, fig. 17.

##### Diagnosis.

 Rs very short; R4+5 five times longer than Rs; R4 nearly as long as R4+5; wing coloration pattern with isolated subapical spot and without spot on R2.

##### Redescription.

 Wing with original coloration pattern of dark clouds visible in medial and distal part; Sc rather long, ending opposite proximal third part of R3; R1 long; cross vein r-r (R2) at its about two lengths before tip of R1; Rs very short; R4+5 five times longer than Rs; R4 nearly as long as R4+5; cross vein r-m just before forks of Rs and of M; distal part R5 and most part of medial veins not preserved, cross vein m-cu about its length behind origin of M3+4; A1 rather long, its distalmost section strongly curved to posterior wing margin.

**Figure 2 A–C.**
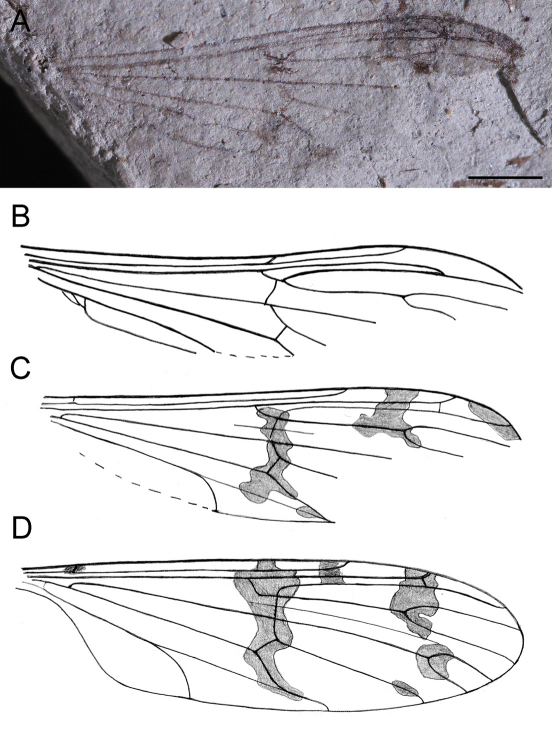
*Ptychoptera deleta* Novák, 1877 **A** photograph of holotype specimen No. NM-P947 **B** original line drawing of wing venation from Novák (1877) **C** line drawing of wing venation (reconstruction) **D** *Ptychoptera agnes* Krzemiński and Zwick, 1993, drawing of wing venation (scale bar represents 1 mm).

##### Dimensions.

Length of wing fragment about 7 mm, probable total length about 7.1 mm; maximum width about 1.8 mm.

##### Holotype.

 No. NM-P947 (imprint of nearly complete wing with medio-apical part missing, posterior wing margin is partially broken, venation well preserved with traces of original pattern of coloration). Specimen is housed in collection of National Museum in Prague, Czech Republic.

##### Age and layer.

Early Miocene (Ottnangian/Karpatian), Cypris Formation (grey claystone) *sensu* Rojík (2004).

##### Discussion.

We provide a re-description of the holotype specimen with several inaccurate characters in wing venation corrected in comparison to the original drawing of Novák, e.g., ending of R1 to anterior wing margin, indication of coloration pattern, A1 basally running parallel to Cu for long distance and ending in posterior wing margin, several missing or present cross-veins. Moreover, we supplement the description by the first photograph of this specimen (unavailable in 1877).

Unfortunately the single diagnostic wing character (M1+2 fork) of the subfamily Ptychopterinae is not preserved. However, the wing venation pattern with very well visible crossvein r-m positioned just before M forking into M1+2 and M3+4, and original color pattern of *Ptychoptera deleta* show considerable similarities to recent species of *Ptychoptera*. These are distinct apomorphic characters present only in this genus within the family Ptychopteridae. The wing venation of *Ptychoptera deleta* is somewhat similar to the recent *Ptychoptera agnes* Krzemiński & Zwick, 1993 described from Hungary (Figs 2C–D). This fact is not surprising due to the Early Miocene age of our fossil (about 18–16 Ma) and considerably high morphological stability of insects (Nel and Prokop 2009; Hörnschemeyer et al. 2009). Finally, our present re-examination of the holotype confirms the great erudition of the Czech pioneer palaeontomologist Dr Ottomar Novák who correctly attributed the fossil species to the otherwise modern genus *Ptychoptera*.

## Supplementary Material

XML Treatment for Ptychoptera

XML Treatment for Ptychoptera deleta
